# Nallo: a Nextflow pipeline for comprehensive human long-read genome analysis

**DOI:** 10.1093/bioinformatics/btag086

**Published:** 2026-02-19

**Authors:** Felix Lenner, Anders Jemt, Lucia Peña Pérez, Ramprasad Neethiraj, Peter Pruisscher, Daniel Schmitz, Annick Renevey, Pádraic Corcoran, Daniel Nilsson, Jesper Eisfeldt, Anna Lindstrand, Valtteri Wirta, Adam Ameur, Lars Feuk

**Affiliations:** Department of Immunology, Genetics and Pathology, Uppsala University, Uppsala, 751 08, Sweden; Genomic Medicine Center Karolinska, Karolinska University Hospital, Stockholm, 171 77, Sweden; Science for Life Laboratory, Department of Microbiology, Tumor and Cell Biology, Karolinska Institutet, Stockholm, 171 77, Sweden; Genomic Medicine Center Karolinska, Karolinska University Hospital, Stockholm, 171 77, Sweden; Science for Life Laboratory, Department of Microbiology, Tumor and Cell Biology, Karolinska Institutet, Stockholm, 171 77, Sweden; Science for Life Laboratory, Department of Microbiology, Tumor and Cell Biology, Karolinska Institutet, Stockholm, 171 77, Sweden; Centre for Inherited Metabolic Diseases, Karolinska University Hospital, Stockholm, 171 77, Sweden; Department of Molecular Medicine and Surgery, Karolinska Institutet, Stockholm, 171 77, Sweden; Science for Life Laboratory, Department of Microbiology, Tumor and Cell Biology, Karolinska Institutet, Stockholm, 171 77, Sweden; Science for Life Laboratory, Department of Microbiology, Tumor and Cell Biology, Karolinska Institutet, Stockholm, 171 77, Sweden; Clinical Genomics Gothenburg, SciLifeLab, Sahlgrenska Academy, University of Gothenburg, Göteborg, 405 30, Sweden; Science for Life Laboratory, Department of Microbiology, Tumor and Cell Biology, Karolinska Institutet, Stockholm, 171 77, Sweden; Department of Immunology, Genetics and Pathology, Uppsala University, Uppsala, 751 08, Sweden; SciLifeLab, Uppsala University, Uppsala, 751 08, Sweden; Science for Life Laboratory, Department of Microbiology, Tumor and Cell Biology, Karolinska Institutet, Stockholm, 171 77, Sweden; Department of Clinical Genetics and Genomics, Karolinska University Hospital, Stockholm, 171 77, Sweden; Department of Molecular Medicine and Surgery, Karolinska Institutet, Stockholm, 171 77, Sweden; Department of Clinical Genetics and Genomics, Karolinska University Hospital, Stockholm, 171 77, Sweden; Department of Molecular Medicine and Surgery, Karolinska Institutet, Stockholm, 171 77, Sweden; Department of Clinical Genetics and Genomics, Karolinska University Hospital, Stockholm, 171 77, Sweden; Genomic Medicine Center Karolinska, Karolinska University Hospital, Stockholm, 171 77, Sweden; Science for Life Laboratory, Department of Microbiology, Tumor and Cell Biology, Karolinska Institutet, Stockholm, 171 77, Sweden; Department of Clinical Genetics and Genomics, Karolinska University Hospital, Stockholm, 171 77, Sweden; Department of Immunology, Genetics and Pathology, Uppsala University, Uppsala, 751 08, Sweden; SciLifeLab, Uppsala University, Uppsala, 751 08, Sweden; Department of Immunology, Genetics and Pathology, Uppsala University, Uppsala, 751 08, Sweden; SciLifeLab, Uppsala University, Uppsala, 751 08, Sweden

## Abstract

**Motivation:**

Long-read sequencing (LRS) is increasingly used for human medical research and clinical diagnostics due to its capacity to generate complete genome information. However, there is a lack of robust and easy-to-use pipelines for comprehensive LRS data analysis.

**Results:**

Here we present Nallo, a Nextflow pipeline for analysis of PacBio and Oxford Nanopore data, with additional support for rare disease research projects. The pipeline detects a wide range of genetic variants, performs genome assembly, and reports CpG methylation. It also enables annotation and ranking of variants based on their predicted functional consequences.

**Availability and implementation:**

Nallo is available from GitHub: https://github.com/genomic-medicine-sweden/nallo

## 1 Introduction

Long-read sequencing (LRS) technologies enable identification of a wide range of genetic variants in the human genome as well as DNA modifications. In recent years, the yield and accuracy of LRS have increased drastically, thereby paving the way for large-scale human LRS projects. Given the current trend toward higher throughput and lower cost per base, it is likely that LRS will replace short-read sequencing for many human genome studies. In fact, this transition has already started, with LRS being increasingly used in clinical diagnostics (Steyaert *et al.* 2024) and sequencing of population cohorts (Gustafson *et al.* 2024, Mahmoud *et al.* 2024).

To fully capitalize on the benefits of human LRS data, it needs to be analyzed with bioinformatic tools capable of extracting different types of genetic events, including single nucleotide variants (SNVs), structural variants (SVs), copy number variants (CNVs), tandem repeats (TRs), and methylation (5mC) signals. Furthermore, the LRS data allow variants to be phased on the two haplotypes and enable *de novo* assembly of a diploid genome sequence (Jarvis *et al.* 2022). Since no single software is capable of providing all relevant analyses, there is a need to collect different tools into a joint workflow, or pipeline. Ideally, the pipeline should be easy to use while producing accurate and reproducible results. Moreover, for clinical research or diagnostics, annotation and ranking of the identified variants are needed in order to facilitate interpretation of the results.

Nallo is a comprehensive pipeline for human LRS analysis. It is developed in Nextflow (Di Tommaso *et al.* 2017), which is increasingly becoming the workflow of choice within the bioinformatics community (Langer *et al.* 2024). Nallo integrates commonly used LRS analysis tools including for alignment, variant calling, and genome assembly, with downstream tools for variant annotation and ranking. All tools are combined into a single pipeline that is under version control and easy to install and run. Nallo is implemented using the nf-core template (Ewels *et al.* 2020), which ensures standardization of the code and facilitates collaborative development. The current version handles data from the two main LRS technology providers, i.e. Oxford Nanopore Technologies (ONT) and PacBio.

## 2 Materials and methods

### 2.1 Overview of the Nallo pipeline

**Figure 1:**
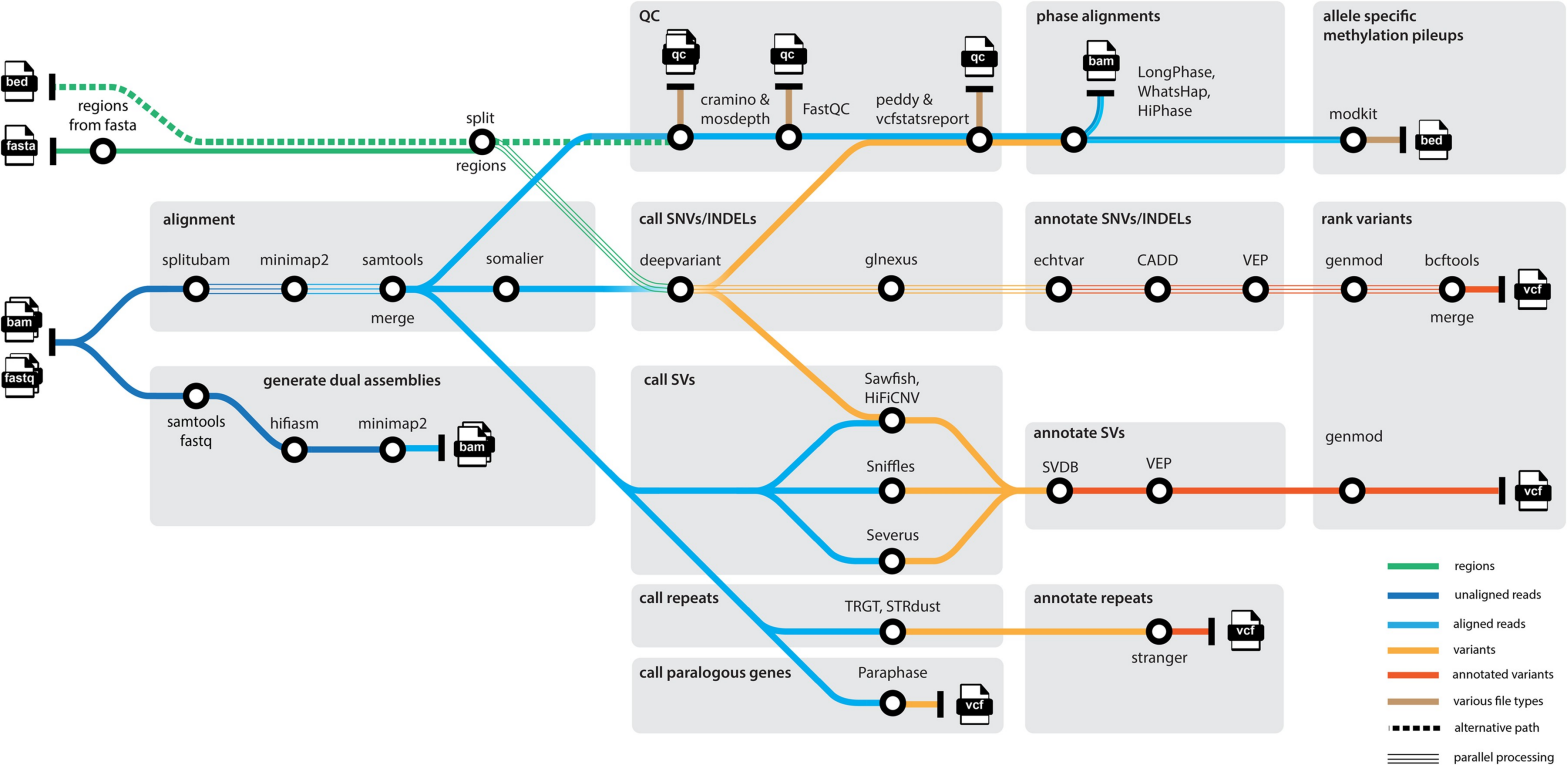
Graphical overview of the Nallo pipeline.

Nallo takes (u)BAM or FASTQ files from any number of (related or unrelated) samples with long sequencing reads as input, i.e. PacBio HiFi reads or base-called ONT reads, and processes them in parallel. The analysis process for a single sample is outlined in Fig. 1, but Nallo also supports family-based analyses, such as trios. Single samples and families can be run simultaneously. Briefly, Nallo starts by aligning the LRS data to the human reference genome, followed by quality control and detection of genetic variants (SNVs, SVs, TRs, CNVs). Then annotation and ranking of potentially deleterious variants is performed, resulting in output files for each sample and combined per family. The alignment files are phased, and if the raw data contain 5mC CpG signals, then this information is preserved and allele-specific methylation pileup data are generated. Furthermore, a *de novo* assembly is created for each individual sample and is aligned to the reference genome. Since some of the analysis tools are preferable for, or restricted to, specific LRS technology, Nallo runs somewhat different tools for PacBio and ONT data, and sub-workflows can be turned on or off at the user’s discretion. Nallo has mainly been evaluated on long-read whole genome sequencing (WGS) data but also works for targeted LRS. The complete list of tools in the current version is available online: https://github.com/genomic-medicine-sweden/nallo.

### 2.2 Implementation and run performance

Nallo is implemented in Nextflow using the nf-core template and guidelines. The implementation is modular, which facilitates adding new tools and tailoring the pipeline for specific needs. It also provides flexibility to bypass analysis steps that are not relevant to a user’s specific application, thereby saving compute time and resources. Moreover, the pipeline can be installed on various types of compute infrastructure. To evaluate the performance, we ran Nallo version 0.5.1 on a ∼32× coverage PacBio trio (HG002, HG003, HG004). Our results show that a complete Nallo analysis, including alignment, variant calling, and *de novo* assembly, consumes about 344 core hours for each sample, completing in 5 hours 52 minutes by executing parallel jobs on a cluster with 68 compute nodes (dual Intel Xeon Gold 6248R, 24 cores @ 3.0 GHz). On the same compute infrastructure, we further executed Nallo on a larger dataset consisting of 192 in-house PacBio samples at 23× average coverage. This analysis was completed in 34 hours. The Nextflow workflow report for this large Nallo run, including resource use for individual tools in the pipeline, is available as a data File 1, available as supplementary data at *Bioinformatics* online.

### 2.3 Annotation, ranking, and filtering of variants

Nallo not only detects genetic variants but can also annotate SNVs, INDELs, SVs, and TRs and reports the results in VCF files. The annotation can be done using public and local databases with variant effect predictions and population frequencies. In addition, Nallo employs a customizable ranking algorithm that allows the user to prioritize variants based on their annotations, such as predicted effects on genes, transcripts, or protein sequences (Stranneheim *et al.* 2021). A filtering step can be applied using criteria such as population allele frequency and genes of interest, thereby generating a shortlist of variants with a high likelihood of being functionally relevant. Annotation, ranking, and filtering of variants are crucial steps in clinical genomics analyses, e.g. when searching for the genetic cause of a rare disease. Since the pipeline was primarily built to replace existing short-read analysis, Nallo currently performs no annotation of methylation signals or of *de novo* assembly results; however, there are external tools that can be used to process the data further, e.g. PAV for assembly-based variant calling (Ebert *et al.* 2021). Furthermore, Nallo is continuously improved, and the addition of more tools is part of the future development plans.

## 3 Conclusions and future perspectives

In addition to Nallo, there exist other workflows for human LRS analysis, including those from the LRS providers ONT and PacBio. The epi2me platform, which is maintained by ONT, includes a Nextflow workflow for human variation (https://github.com/epi2me-labs/wf-human-variation). PacBio has developed its own solution using the workflow description language (WDL) (https://github.com/PacificBiosciences/HiFi-human-WGS-WDL). There have also been open-source efforts within the nf-core community, such as nf-core/nanoseq (https://github.com/nf-core/nanoseq) and nf-core/pacvar (Jain and Clelland 2025). Contrary to the above pipelines, Nallo is designed to handle both ONT and PacBio data. Furthermore, Nallo has additional features, such as the option to run multiple SV callers, perform single-sample or family-based analyses, and provide annotation and ranking to support the analysis of rare disease variants. However, all pipelines developed within or following the nf-core template can take advantage of the collaborative environment.

Nallo was originally developed for the identification of pathogenic genetic variants in rare disease patients (Eisfeldt *et al.* 2024), but the pipeline can also be used also for projects related to complex disease, pharmacogenomics, and population genomics. Specific application areas could benefit from additional analysis tools, e.g. for HLA and CYP genes, which we intend to include in future releases. Tools for additional quality control and cleanup, such as Breakinator to detect foldback artifacts in ONT data, would also improve Nallo further (Heinz *et al.* 2025). Another limitation with Nallo is that the current version is restricted to germline variation. Although Nallo could be expanded to somatic variation detection and paired tumor-normal analysis in cancer, such analyses are not yet supported.

Since bioinformatic analysis of LRS data is rapidly evolving, we anticipate that Nallo will be regularly updated as new tools and improved software versions become available. In this context, the ability of Nextflow to handle different execution platforms is a big advantage, thereby facilitating updates on local machines, clusters, and cloud environments. Nallo also lays a foundation for larger international collaborations. Having a common framework for LRS analysis facilitates data sharing and reduces batch effects between projects and institutes. In this way, our proposed workflow can play an important role in large-scale projects aiming to better understand the human genome and its role in health and disease.

## Supplementary Material

btag086_Supplementary_Data

## References

[btag086-B1] Di Tommaso P , Chatzou M, Floden EW et al Nextflow enables reproducible computational workflows. Nat Biotechnol 2017;35:316–9. 28398311 10.1038/nbt.3820

[btag086-B2] Ebert P , Audano PA, Zhu Q et al Haplotype-resolved diverse human genomes and integrated analysis of structural variation. Science 2021;372:eabf7117. 10.1126/science.abf7117 PMC8026704 33632895

[btag086-B3] Eisfeldt J , Ameur A, Lenner F et al A national long-read sequencing study on chromosomal rearrangements uncovers hidden complexities. Genome Res 2024;34:1774–84. 39472022 10.1101/gr.279510.124 PMC11610602

[btag086-B4] Ewels PA , Peltzer A, Fillinger S et al The nf-core framework for community-curated bioinformatics pipelines. Nat Biotechnol 2020;38:276–8. 32055031 10.1038/s41587-020-0439-x

[btag086-B5] Gustafson JA , Gibson SB, Damaraju N et al High-coverage nanopore sequencing of samples from the 1000 Genomes Project to build a comprehensive catalog of human genetic variation. Genome Res 2024;34:2060–73. 10.1101/gr.279273.124 PMC11610458 39358015

[btag086-B6] Heinz JM , Meyerson M, Li H. Detecting foldback artifacts in long-reads. BMC Genomics 2026;27:144. 41495659 10.1186/s12864-025-12492-y PMC12870357

[btag086-B7] Jain T , Clelland C. nf-core/pacvar: a pipeline for analyzing long-read PacBio whole genome and repeat expansion sequencing data. Bioinformatics 2025;41:btaf116. 10.1093/bioinformatics/btaf116 PMC11964484 40098241

[btag086-B8] Jarvis ED , Formenti G, Rhie A et al; Human Pangenome Reference Consortium Semi-automated assembly of high-quality diploid human reference genomes. Nature 2022;611:519–31. 36261518 10.1038/s41586-022-05325-5 PMC9668749

[btag086-B9] Langer BE et al Empowering bioinformatics communities with Nextflow and nf-core. Genome Biol. 2025:26:228. 10.1186/s13059-025-03673-9 PMC12309086 40731283

[btag086-B10] Mahmoud M , Huang Y, Garimella K et al Utility of long-read sequencing for all of us. Nat Commun 2024;15:837. 38281971 10.1038/s41467-024-44804-3 PMC10822842

[btag086-B11] Steyaert W , Sagath L, Demidov G, Solve-RD DITF-EpiCARE et al Unravelling undiagnosed rare disease cases by HiFi long-read genome sequencing. Genome Res 2025;35:755–68. 10.1101/gr.279414.124 PMC12047270 40138663

[btag086-B12] Stranneheim H , Lagerstedt-Robinson K, Magnusson M et al Integration of whole genome sequencing into a healthcare setting: high diagnostic rates across multiple clinical entities in 3219 rare disease patients. Genome Med 2021;13:40. 33726816 10.1186/s13073-021-00855-5 PMC7968334

