# Supplementary material for: Nallo: a Nextflow pipeline for comprehensive human long-read genome analysis
Source: Bioinformatics. 2026 Feb 19;42(3):btag086. doi: 10.1093/bioinformatics/btag086 (PMC12988770; doi:10.1093/bioinformatics/btag086)
Supplement: btag086_Supplementary_Data [file btag086_supplementary_data.zip › Supplementary file 1.html]

[distracted\_engelbart] Nextflow Workflow Report


Nextflow Report


- Summary
- Resources
- Tasks

[distracted\_engelbart]

# Nextflow workflow report

## `[distracted_engelbart]`

Workflow execution completed successfully!

Run times
:   18-Sep-2025 22:27:22 - 20-Sep-2025 07:52:00
    (duration: **1d 9h 24m 38s**)

194379 succeeded

0 cached

0 ignored

0 failed (0 retries)

Nextflow command
:   ```
    nextflow run nallo/workflow/genomic-medicine-sweden-nallo_0.7.1/0_7_1/main.nf -c nallo/configs/configs_0.7.1/nallo_config_0.7.1_mod.config --input 192samples_mod.csv -profile singularity --outdir results -with-report report.html -with-timeline timeline.html --publish_dir_mode copy -w work --alignment_processes 1
    ```

CPU-Hours
:   `73'382.2`

Workflow profile
:   singularity

Nextflow version
:   version 25.04.7, build 5955 (08-09-2025 13:29 UTC)

## Resource Usage

These plots give an overview of the distribution of resource usage for each process.

#### CPU

- Raw Usage
- % Allocated

#### Memory

- Physical (RAM)
- Virtual (RAM + Disk swap)
- % RAM Allocated

#### Job Duration

- Raw Usage
- % Allocated

#### I/O

- Read
- Write

## Tasks

This table shows information about each task in the workflow. Use the search box on the right
to filter rows for specific values. Clicking headers will sort the table by that value and
scrolling side to side will reveal more columns.

Values shown as:

Human readable
Raw values

(tasks table omitted because the dataset is too big)

Generated by Nextflow, version 25.04.7
